# Blood Pressure Response to the Head-Up Tilt Test in Benign Paroxysmal Positional Vertigo

**DOI:** 10.3390/jcm12247725

**Published:** 2023-12-16

**Authors:** Moon-Jung Kim, Guil Rhim

**Affiliations:** 1 Department of Laboratory Medicine, Myongji Hospital, Hanyang University Medical Center, Goyang 10475, Republic of Korea; kimmunj@gmail.com; 2 Department of Otorhinolaryngology, One Otorhinolaryngology Clinic, Paju 10924, Republic of Korea

**Keywords:** benign paroxysmal positional vertigo, head-up tilt test, blood pressure, vestibulosympathetic reflex, autonomic nervous system

## Abstract

The vestibular organ is involved in controlling blood pressure through vestibulosympathetic reflexes of the autonomic nervous system. This study aimed to investigate the effect of benign paroxysmal positional vertigo (BPPV) on blood pressure control by the autonomic nervous system by observing changes in blood pressure before and after BPPV treatment using the head-up tilt test (HUTT). A total of 278 patients who underwent the HUTT before and after treatment were included. The HUTT measured blood pressure repeatedly on the day of diagnosis and the day of complete recovery, and the results were analyzed using repeated measures analysis of variance. Regarding the difference in the systolic blood pressure of patients with BPPV, the blood pressure at 1, 2, and 3 min in the upright position after complete recovery was significantly lower than before treatment (*p* = 0.001, *p* = 0.001, and *p* = 0.012, respectively). Blood pressure at 1 and 2 min in the diastolic blood pressure of patients with BPPV in the upright position after complete recovery was significantly lower than before treatment (*p* = 0.001 and *p* = 0.034, respectively). This study shows that BPPV increases blood pressure during the initial response to standing in the HUTT.

## 1. Introduction

Baroreceptors and vestibulosympathetic reflexes (VSRs) play important roles in regulating blood pressure in the autonomic nervous system. Studies have been conducted on the role of vestibular organs connected to the VSR in controlling blood pressure [1]. In a study that observed changes in blood pressure and pulse rate through horizontal linear acceleration that activated the otolith organ, systolic blood pressure increased by 7–9 mmHg and diastolic blood pressure increased by 3–5 mmHg. In addition, patients with bilateral reduction in vestibular input show much smaller cardiovascular alterations during acceleration than those with normal vestibular function [2]. Cardiovascular control is associated with breathing. In a study of cold vestibular caloric simulation, heart rate and blood pressure variabilities increased during spontaneous breathing; however, during paced breathing, no consistent effect on heart rate variability or blood pressure variability was evident. This change was not observed in the labyrinthine-defective patient [3]. Changes in posture require rapid cardiovascular adjustments to maintain blood pressure and volume distribution. In healthy volunteers, heart rate is accelerated by head drops that occur within 500–600 ms of a beat; however, no rapid effects are observed in patients with vestibular defects [4,5].

These data suggest that vestibular stimulation can produce cardiovascular responses in humans and support the hypothesis that the vestibular system contributes to maintaining stable blood pressure during movement and changes in posture. Recent studies have indicated that otolithic organ dysfunction affects the autonomic nervous system. Studies have indicated that male patients in the absent vestibular-evoked myogenic potential (VEMP) group have a significant decrease in diastolic blood pressure 1 min after active standing up [6], indicating that utricular dysfunction is associated with orthostatic hypotension (OH). OH is associated with high baseline systolic blood pressure, heart failure, and unilateral VEMP abnormalities [7]. Studies have also indicated that the ratio of arrhythmia or heart rate variability markers is higher in patients with benign paroxysmal positional vertigo (BPPV) than in control groups [8,9]. OH, which accompanies BPPV, affects the recurrence of BPPV [10]. The above findings also show that labyrinthine defective patients have a lower cardiovascular response to changes in the body’s position, and abnormalities in otolith organs affect changes in blood pressure. This study aimed to determine the effects of BPPV on blood pressure control in the autonomic nervous system by observing changes in blood pressure before and after BPPV treatment using the head-up tilt test (HUTT). BPPV increases blood pressure during the initial response to standing in the HUTT.

## 2. Materials and Methods

### 2.1. Study Subjects

This prospective study included patients diagnosed with BPPV who visited the primary otorhinolaryngology clinic with vertigo symptoms from December 2021 to April 2023. When a patient who visited the hospital due to a symptom of vertigo showed any abnormal finding in the medical history assessment, general otolaryngological examination, and physical examination using a Frentzel glass, the following tests were carried out.

### 2.2. Test Protocol

None of the patients had consumed alcohol within 12 h of the test, and drugs for high blood pressure and diabetes, excluding stabilizers, were taken as usual. All patients were tested without the placement of intravenous or intra-arterial lines. After resting for 15 min in a quiet and comfortable room, the patient was laid on a test table with a footplate and restrained using two Velcro straps placed around the legs and waist. Regarding the sequence of the test, blood pressure and pulse rate were observed by automated cuff measurements in the right arm twice at 1 min intervals after resting for 3 min in a lying position; the table was raised to 70° for 30 s and then blood pressure and pulse rate were measured three times at 1 min intervals. Furthermore, all patients underwent positioning tests using videonystagmography. After the diagnosis of BPPV, repositioning maneuvers were performed twice a week; treatments were stopped when symptoms disappeared, and the nystagmus resolved according to a video Frentzel glass examination. The HUTT was performed again on the day of the completion of treatment. All patients were diagnosed and treated by a physician.

### 2.3. Diagnosis and Treatment

Based on the Bárány Society’s diagnostic criteria for BPPV [11], the distribution characteristics of different BPPV types were as follows: 117 (42.0%) patients had horizontal canal BPPV—canalolithiasis, 86 (30.9%) patients had posterior canal BPPV—canalolithiasis, 31 (11.0%) patients had horizontal canal BPPV—cupulolithiasis, 8 (2.8%) patients had anterior canal BPPV—canalolithiasis, and 36 (12.9%) patients had multiple canal BPPV. Dix-Hallpike, supine roll, and supine straight head-hanging tests were performed to determine lesion locations. For posterior canal BPPV, the Dix-Hallpike test was positive if nystagmus was recorded with appropriate positioning, latency, duration, and fatigability. Lateral canal BPPV was diagnosed by horizontal direction-changing positional nystagmus concurrent with vertigo triggered by the supine roll test. Lateral canal BPPV was classified as canalolithiasis or cupulolithiasis according to the direction of the nystagmus, as horizontal geotropic and apogeotropic nystagmus, respectively. For horizontal canal BPPV- canalolithiasis, in the supine position, transient lying-down nystagmus frequently occurs. In the right-ear-down position, horizontal nystagmus toward the right occurs after a brief period and then decays and stops within 1 min. In the left-ear-down position, horizontal nystagmus toward the left occurs after a brief period and then decays and stops within 1 min. Down-beating nystagmus is accompanied by a less-pronounced torsional component to the affected side suggested anterior semicircular canal-BPPV based on supine straight head-hanging tests or the Dix-Hallpike test [11,12]. 

For treatment, the modified Epley method was used in the case of canalolithiasis of the posterior semicircular canal, the Barbecue rotation method was used in the case of canalolithiasis of the lateral semicircular canal, and the Appiani and Gufoni methods were used in the case of lateral canal cupulolithiasis. The Appiani maneuver rotates the head upward in the third therapeutic position and aims to reposition the canalith within the anterior arm or the cupulolith on the canal side of the cupula. On the other hand, the original Gufoni maneuver, introduced by Mauro Gufoni in 1998, should be performed in cases where the cupulolith is attached to the utricular side of the cupula; the patient lies down quickly on the side with the unaffected ear and remains in this position for 1 to 2 min until the evoked nystagmus subsides. The head is then quickly rotated 45° toward the floor. The Yacovino method was used in the case of canalolithiasis of the anterior semicircular canal. During the course of treatment, any stabilizers were not taken.

### 2.4. Patient Data and Variables

**Figure 1:**
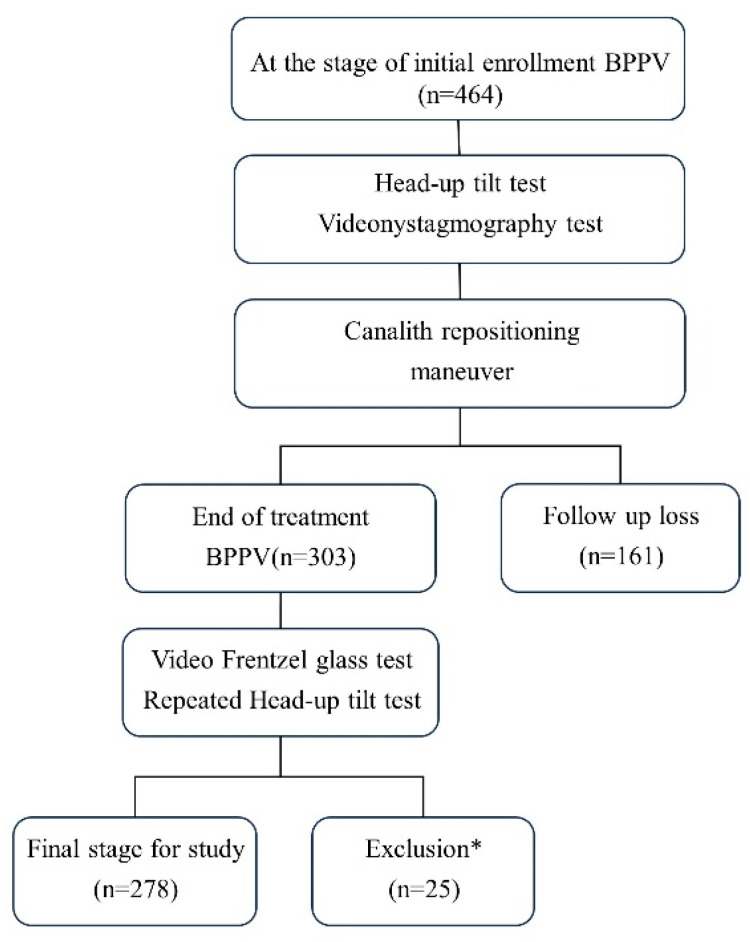
Flowchart of the study design. * For the exclusion criteria, see the Section 2 Materials and Methods.

During the study period, 464 patients were diagnosed with BPPV and 278 were included in this study. Patients aged < 20 years, with chronic otitis media, cochlear hydrops, Meniere’s disease, varicose veins, OH, postural orthostatic tachycardia syndrome, and those who had not been followed up were excluded (Figure 1). Age, sex, hypertension, and diabetes were assessed using patients’ medical records and questionnaires. In the HUTT, systolic and diastolic blood pressure was measured at 1 min intervals in the supine position and three times at 1 min intervals in the upright position. This study was approved by the Myongji Hospital Institutional Review Board No. 2023-08-007 and, therefore, has been carried out in accordance with the ethical standards of the 1964 Declaration of Helsinki and its subsequent amendments. Written informed consent was obtained from all participants before inclusion in the study.

### 2.5. Statistical Analysis

Data are presented as arithmetic means with standard deviations and 95% confidence intervals. This study used repeated measures analysis of variance (ANOVA). The Kolmogorov–Smirnov test was used to test for normality, and when required, the probability of a non-normal distribution of the analyzed data was assessed using the Friedman test. Box’s test was used to verify equal variance, and Mauchly’s sphericity test verified the ε value of Greenhouse-Geisser univariate tests. Statistical analyses were performed using IBM SPSS ver. 19.0 (IBM Corp., Armonk, NY, USA). Statistical significance was set at *p* < 0.05.

## 3. Results

The average age (SD) of the 278 participants was 52.17 (13.73) years (20–88 years) and 192 (69%) of them were female. The prevalence rates of hypertension and diabetes in the study participants were 27% and 13%, respectively. The average treatment period was 7.31 days (Table 1).

**Figure 2:**
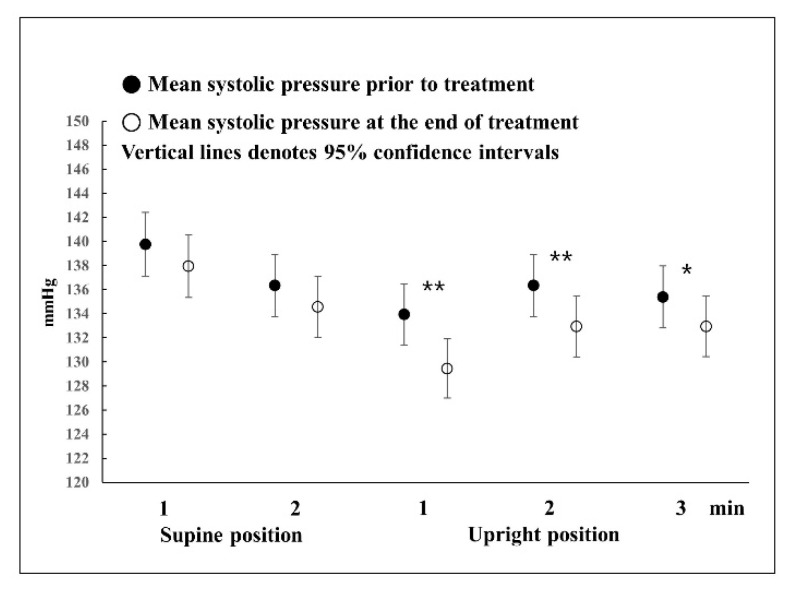
Differences in systolic blood pressure before and after BPPV treatment. Repeated measures ANOVA with Greenhouse-Geisser correction was used to analyze the effect of time during the HUTT on systolic blood pressure before and after treatment (F = 51.053, *p* < 0.001, ε = 0.734). There were statistically significant differences in systolic blood pressure at 1, 2, and 3 min before and after treatment in the upright position. * *p* < 0.05, ** *p* < 0.01.

Regarding the difference in systolic blood pressure before and after BPPV treatment (Figure 2), blood pressure at 1, 2, and 3 min in the upright position after complete recovery was significantly lower than before treatment (*p* = 0.001, *p* = 0.001, and *p* = 0.012).

**Figure 3:**
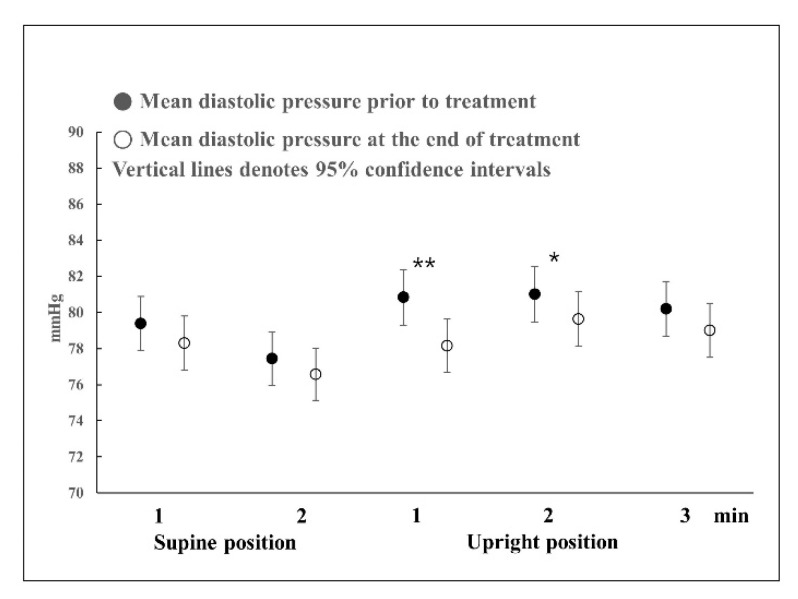
Differences in diastolic blood pressure before and after BPPV treatment. Repeated measures ANOVA with Greenhouse-Geisser correction was used to analyze the effect of time during the HUTT on diastolic blood pressure before and after treatment (F = 34.704, *p* < 0.001, ε = 0.655). There were statistically significant differences in diastolic blood pressure at 1 and 2 min before and after treatments in the upright position. * *p* < 0.05, ** *p* < 0.01.

Regarding the difference in diastolic blood pressure before and after BPPV treatment (Figure 3), blood pressure at 1 and 2 min in the upright position after complete recovery was significantly lower than before treatment (*p* = 0.001 and *p* = 0.034, respectively).

**Figure 4:**
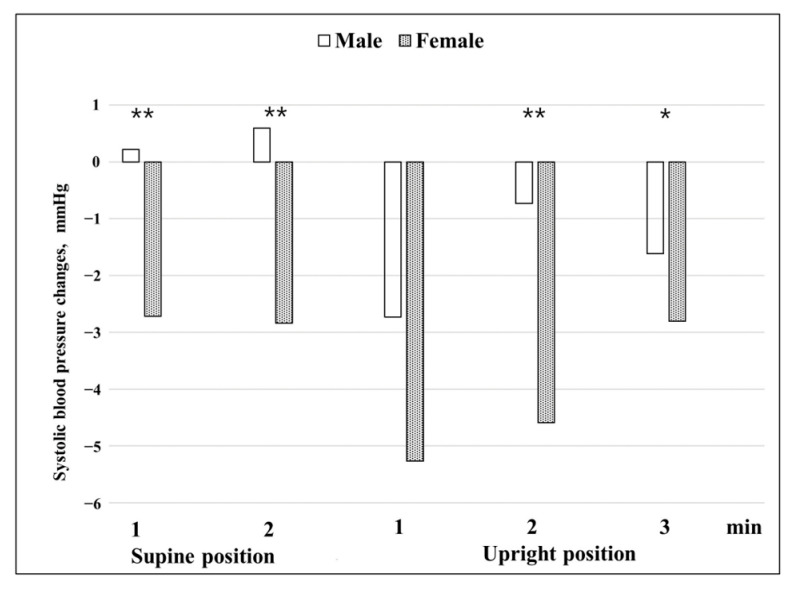
Changes in systolic blood pressure before and after treatment according to sex. Blood pressure before treatment was the baseline. Repeated measures ANOVA with Greenhouse-Geisser correction was used to analyze the effect of time during the HUTT on systolic blood pressure before and after treatment according to sex (F = 49.358, *p* < 0.001, ε = 0.735). In the test of between-subject effects, sex: F = 7.305, *p* = 0.007. * *p* < 0.05, ** *p* < 0.01.

**Figure 5:**
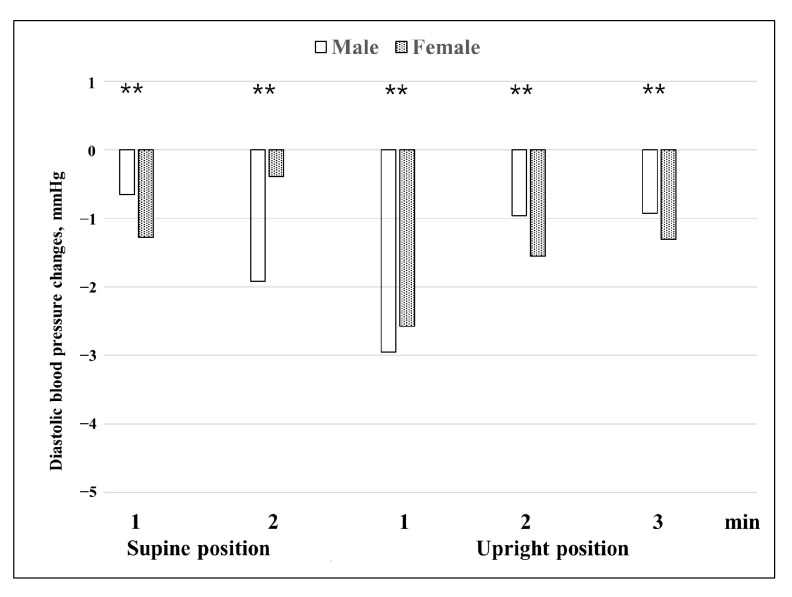
Changes in diastolic blood pressure before and after treatment according to sex. Blood pressure before treatment was the baseline. Repeated measures ANOVA with Greenhouse-Geisser correction was used to analyze the effect of time during the HUTT on diastolic blood pressure before and after treatment according to sex (F = 35.049, *p* < 0.001, ε = 0.654). In the test of between-subject effects, sex: F = 26.024, *p* = 0.000. ** *p* < 0.01.

When the difference in changes in blood pressure before and after BPPV treatment between the sexes was examined (Figure 4 and Figure 5), it was observed that the amount of change in systolic blood pressure in females was significantly greater than in males at 1 and 2 min in the supine position and at 2 and 3 min in the upright position (*p* = 0.005, *p* = 0.007, *p* = 0.006, and *p* = 0.016). Females’ systolic blood pressure decreased more than 2.5 mmHg compared to before treatment at all times and showed a particularly large change in the upright position at 1 and 2 min. In males, the changes in systolic blood pressure were almost unchanged within 1.5 mmHg except at 1 min in the upright position. The amount of change in diastolic blood pressure was a 0.4–3 mmHg decrease at all times after complete recovery in both males and females. Blood pressure decreased significantly more in males than in females at 2 min in the supine position and at 1 min in the upright position (*p* = 0.000 and *p* = 0.000), and decreased significantly more in females than in males at 1 min in the supine position and at 2 and 3 min in the upright position (*p* = 0.000, *p* = 0.000, and *p* = 0.000, respectively).

## 4. Discussion

In this study, three implications were found: (1) even in the presence of BPPV, blood pressure was normal in the supine position; (2) even in the presence of BPPV, blood pressure showed a pattern similar to a normal response in the HUTT; and (3) BPPV stimulates the otolith organ and activates the VSR, thus increasing blood pressure during the early stages of the HUTT.

Most studies that use the HUTT have been on syncope, and there are few detailed results on normal responses. Furthermore, most HUTTs are performed until symptoms appear in a tilted state or for up to 45 min for the diagnosis of syncope [13]. Therefore, only a few studies have mentioned up to 5 min after the onset of the HUTT. According to the findings of several studies on normal responses, in the upright position, the diastolic blood pressure increases slightly and systolic blood pressure decreases slightly before reaching a stable state at 2 min [14,15,16]. In this study, in the HUTT in the supine position, there were also no changes in blood pressure before and after treatment. Systolic blood pressure dropped at 1 min in the upright position and then increased slightly at 2 or 3 min, but the systolic blood pressure pattern was the same before and after treatment. However, the systolic blood pressure before treatment decreased to less than that after complete recovery. Diastolic blood pressure showed slightly different responses before and after treatment. Before treatment, diastolic blood pressure increased at 1 min in the upright position, causing a large change in blood pressure, and then returned to a level similar to that of blood pressure in the supine position at 2 or 3 min. Regarding changes in blood pressure after complete recovery, the increase rate was lower than that of the blood pressure response before treatment; thus, blood pressure slowly increased until 2 min in the upright position and normalized thereafter. That is, in the presence of BPPV, blood pressure showed a rapid response in the upright position, such that systolic and diastolic blood pressure showed the largest change at 1 min; however, after complete recovery, such responses were not observed. When blood pressure was measured after sufficient rest, there was no difference in blood pressure in the supine position before and after treatment; therefore, it can be said that blood pressure at rest was normal even in the presence of BPPV. Meanwhile, the symptoms of vertigo or dizziness could trigger acute stress that increases blood pressure, which is not linked to vestibular stimulation. Although the difference in blood pressure in the supine position before and after treatment was not statistically significant, it can be seen that the blood pressure before treatment was slightly higher than after treatment. The possibility cannot be excluded that the increase in blood pressure was caused by acute stress without stimulation of the vestibular organ in the supine position. Excitement or depression could affect blood pressure. A previous study indicated a significant relationship between low blood pressure and increased depressive symptomatology scored using the Center for Epidemiologic Studies Depression Scale [17]. Systolic hypotensive subjects scored a Center for Epidemiologic Studies Depression Scale mean of 12.07 ± 0.67 compared to 8.99 ± 0.95 for normotensives (*p* < 0.01). Regression analyses supported these findings when controlling for confounders such as gender, age, and the use of antihypertensive medications. However, Menant et al. [18] reported that participants were classified as having dizziness based on the Dizziness Handicap Inventory. Participants completed health questionnaires and underwent assessments of psychological well-being, lying and standing blood pressure, and vestibular function. Lying and standing blood pressure were not significantly associated with dizziness handicap severity.

However, during standing, when the VSR is overworking, the sympathetic nerve works toward excitation to increase the overall blood pressure; for this reason, it is believed that there is a statistically significant difference in blood pressure before and after treatment.

When differences in blood pressure changes before and after treatment between males and females were examined (Figure 4 and Figure 5), both systolic and diastolic blood pressures showed changes in a V shape around 1 min in the upright position. This means that in the presence of BPPV, blood pressure is most affected at 1 min in the upright position, and it can be seen that the VSR is affected by BPPV. In general, the effects of vestibular organs on the cardiovascular system occur within one or two heartbeats after rapid head movement, and the VSR has a latency of approximately 400–500 ms [5,19]. From the name of VSR, it can be seen that vestibular autonomic nerve reflection mainly affects the sympathetic nervous system. VSR suggests that otolith signals are important and operate more rapidly than baroreceptors in response to changes in blood pressure caused by body movement [1,2,3]. The otolith is a linear accelerometer that measures gravitational and inertial acceleration [20]. Raphan et al. [21] reported that VSR modulates blood pressure and heart rate in an oscillating system by manipulating the parameters of the baroreflex feedback and the signals that maintain the oscillations.

Furthermore, it appears that in the case of BPPV, the autonomic nervous system is more vulnerable in females; therefore, the amount of change in blood pressure is generally higher in females than in males. Our findings that the difference in blood pressure changes before and after treatment between males and females (Figure 4 and Figure 5) are consistent with previously reported data obtained using shorter HUTT protocols. Braune et al. [22] reported that sex had a significant influence on changes in blood pressure during passive HUTTs. Females showed more changes in blood pressure than males during the HUTT. In the orthostatic maneuvers, males showed lower blood pressure values compared with females. With increasing age, this difference became even more evident. The data suggest that the blood pressure responses to orthostatic stressors are attenuated with increasing age in contrast to the preserved blood pressure activation during non-orthostatic challenges. The reasons for the different impacts of age on blood pressure regulation remain to be identified. Some studies have found that there are sex effects on cardiovascular autonomic function during passive HUTTs [23,24]. In young women, variability in resting sympathetic nerve activity (SNA) is similar to that seen in men, but the ‘balancing’ mechanisms are strikingly different. Women exhibit greater β-adrenergic vasodilatation compared with men, which minimizes the pressor effects of a given level of SNA. In healthy men, the variability in SNA is balanced by variability in cardiac output and vascular adrenergic responses, such that blood pressure remains similar. Loss of estrogen with menopause in women appears to be linked mechanistically with a decrease in β-adrenergic vasodilatation and that suggest that β-adrenergic receptor sensitivity to noradrenaline is reduced in postmenopausal women. Another study observed that in men, mean blood pressure at rest (*p* = 0.001 systolic and *p* = 0.004 diastolic) and during HUTT (*p* = 0.001 systolic and *p* = 0.001 diastolic), mean total peripheral resistance at rest (*p* = 0.034), and mean stroke volume during HUTT (*p* = 0.001) were significantly higher. Particularly in older women, orthostatic changes in heart rate and diastolic blood pressure, the deep breathing ratio, and the Valsalva ratio become attenuated with age [24]. Regarding the average age of females, 52.5 years in this study, the difference in the change in blood pressure between the sexes was partially explained. A previous study on the otolith organ and the autonomic nervous system by Aoki et al. [6] classified the otolith organ according to the VEMP responses and reported that in the case of males, when the VEMP response was absent, the diastolic blood pressure dropped significantly compared to other groups at 1 min of active standing, and that in the case of the asymmetry and normal VEMP groups, the systolic blood pressure increased at 1 min of standing. The study reported that, in the case of females, there was no difference between the absent and other groups. Compared to our study, the study by Aoki et al. was an active standing test study, while our study was a passive standing test study; however, the most significant results were shown at 1 min of standing in both studies. Therefore, it can be seen that the time in which VSR has the largest effect is 1 min of standing. Furthermore, the fact that the responses of males and females differed can be considered a common point.

Kim et al. [7] reported that in a HUTT using a finometer, OH was associated with high baseline systolic blood pressure, heart failure, and unilateral ocular VEMP abnormalities. The n1 latency of VEMP was negatively correlated with maximal changes in systolic blood pressure and utricular dysfunction related to OH. In a similar study, Kim et al. [10] reported that when BPPV was present, the group without accompanying OH had fewer recurrences. In a study on the autonomic nervous system and BPPV associated with the heart, Günlü et al. [9] reported that Holter monitoring detected abnormal rhythms in 41 (63%) of the patients in the study group and three (6.2%) of the patients in the control group. Yıldırım et al. [8] reported that the Tp-e and Tp-e/QTc ratios were significantly higher in patients compared to controls. These findings suggest that the risk of ventricular arrhythmia is higher in patients with BPPV. The findings of the previous studies mentioned above indicate that abnormalities in the otolith organs are related to the autonomic nervous system. Patients with vestibular defects have a lower response to postural changes than those with normal vestibular function [3,4,5]. However, in general, when the otolith organ is stimulated, the responses of the sympathetic nerves are diverse. Ray et al. [25] reported that yaw head rotation, which stimulates horizontal semicircular canals, elicits a sympathetic nerve response. Yaw head rotation did not significantly change sympathetic nerve activity or mean arterial pressure. In another study, age-related VSR experiments showed that head-down rotation performance during simultaneous orthostatic stress further increased total muscle sympathetic nerve activity in young subjects, but not in older subjects. Older subjects consistently showed significant hypotension during head-down rotations [19]. VSRs differ from responses triggered by the unloading of cardiovascular receptors, such as baroreceptors and cardiopulmonary receptors, as they can be elicited before a change in blood distribution occurs in the body [1]. Therefore, it can be seen that in various studies in which VSR works, different results are produced according to various conditions.

This is one of the studies on the various autonomic nervous system responses of the otolith organ and helps verify that BPPV affects the autonomic nervous system through VSR and increases blood pressure during the initial response to the HUTT. Compared to other studies showing that patients with vestibular defects have small changes in blood pressure due to stimuli, such as changes in posture, this study did not show that BPPV reduces vestibular function. Among the participants in this study, patients with ear disease were excluded; however, patients with diabetes, high blood pressure, or heart disease that affected the results of the HUTT could not be excluded. Based on these findings, a follow-up study is needed to identify the potential risk factors for blood pressure response in the presence of BPPV. 

## 5. Conclusions

This study showed that BPPV increased blood pressure during the initial response to standing in the HUTT.

## Figures and Tables

**Table 1:** Study characteristics.

	Number of Subjects (%)	Age (Mean ± SD)	HIBP (%)	DM (%)	Duration of Treatment (Days) (Mean ± SD)
Male	86 (31)	51.5 ± 14.1 (20–86 y)	23 (27)	14 (16)	7.1 ± 3.2
Female	192 (69)	52.5 ± 13.6 (22–88 y)	52 (27)	22 (12)	7.4 ± 3.9
Total (%)	278	52.2 ± 13.7	75 (27)	36 (13)	7.3 ± 3.6

SD, standard deviation; HIBP, hypertension; DM, diabetes mellitus.

## Data Availability

Data presented in this study are available upon request from the corresponding author upon reasonable request.
